# Emerging Optical Materials in Sensing and Discovery of Bioactive Compounds

**DOI:** 10.3390/s21175784

**Published:** 2021-08-27

**Authors:** Raquel Vaz, Beatriz Valpradinhos, Manuela F. Frasco, Maria Goreti F. Sales

**Affiliations:** 1 BioMark@UC, Faculty of Sciences and Technology, University of Coimbra, 3030-790 Coimbra, Portugal; rsrvaz@student.uc.pt (R.V.); goreti.sales@eq.uc.pt (M.G.F.S.); 2 CEB—Centre of Biological Engineering, University of Minho, 4710-057 Braga, Portugal; b.valpradinhos@gmail.com; 3 BioMark@ISEP, School of Engineering, Polytechnic Institute of Porto, 4249-015 Porto, Portugal

**Keywords:** biosensors, optical detection, aquatic biotoxins, drug discovery, bioactive compounds

## Abstract

Optical biosensors are used in numerous applications and analytical fields. Advances in these sensor platforms offer high sensitivity, selectivity, miniaturization, and real-time analysis, among many other advantages. Research into bioactive natural products serves both to protect against potentially dangerous toxic compounds and to promote pharmacological innovation in drug discovery, as these compounds have unique chemical compositions that may be characterized by greater safety and efficacy. However, conventional methods for detecting these biomolecules have drawbacks, as they are time-consuming and expensive. As an alternative, optical biosensors offer a faster, simpler, and less expensive means of detecting various biomolecules of clinical interest. In this review, an overview of recent developments in optical biosensors for the detection and monitoring of aquatic biotoxins to prevent public health risks is first provided. In addition, the advantages and applicability of these biosensors in the field of drug discovery, including high-throughput screening, are discussed. The contribution of the investigated technological advances in the timely and sensitive detection of biotoxins while deciphering the pathways to discover bioactive compounds with great health-promoting prospects is envisaged to meet the increasing demands of healthcare systems.

## 1. Introduction

Biotoxins are biological toxic substances produced by various organisms, namely, animals (animal toxins), plants (phytotoxins), and microorganisms (mycotoxins, cyanotoxins, and toxins of dinoflagellates and diatoms). These chemical substances are produced in stress, predation, and defense situations [1]. In particular, aquatic natural toxins, which include freshwater and marine sources (such as algae, coelenterates, reef fish, dinoflagellates, and cyanobacteria), pose a serious threat to public health through exposure by inhalation of aerosolized toxins, dermal absorption, or transmission through the food chain. Ingestion of biotoxins leads to gastrointestinal, neurological, and cardiovascular syndromes, which in severe cases can result in death. To prevent poisoning from aquatic biotoxins, seafood and shellfish meat must be monitored, and the levels of toxin-producing microorganisms in the water must be detected before marketing. In addition to human health, environmental poisoning can also cause fatalities in fish, birds, and marine mammals [2]. For example, microcystins produced by cyanobacteria can kill animals living in eutrophic freshwater ecosystems or accumulate in mollusks, fish, and crayfish consumed by humans [3]. Additionally, increase in harmful algal blooms, possibly due to rising ocean temperatures, anthropogenic pressures, and increasing coastal eutrophication, contaminates various water sources, especially drinking water, and causes poisoning in animals and humans [4].

Aquatic biotoxins and their analogues can be classified according to their chemical structure, source of origin, or mechanism of toxicity (Table 1). In the first classification, biotoxins are usually alkaloids, polyethers, or peptides. For example, tetrodotoxin and saxitoxin are marine alkaloids and are neurotoxins [5]. Regarding the source of the toxin, there are, for example, microcystins from cyanobacteria [6], conotoxin from species of the genus *Conus* [7], tetrodotoxin from pufferfish [8], and dinophysistoxins from species of the genus *Dinophysis* [9]. The mechanism of toxicity includes the human syndromes that biotoxins cause: diarrhetic shellfish poisoning (DSP), caused by okadaic acid, dinophysistoxins, pectenotoxin, and yessotoxin; paralytic shellfish poisoning (PSP), caused by saxitoxin; amnesic shellfish poisoning (ASP), caused by domoic acid; azaspiracid shellfish poisoning (AZP), caused by azaspiracid; ciguatera fish poisoning (CFP) due to ciguatoxins; and neurotoxic shellfish poisoning (NSP) due to brevetoxins [5,10].

Although toxins are usually considered harmful, many aquatic biotoxins hold great pharmaceutical potential, the value of which has only been recognized in recent years with the growing need for new drugs. Available drugs are effective in only one-third of diseases, and pathogens have developed resistance to them. Therefore, new biologically active compounds with improved therapeutic activities should be considered. For example, the aquatic biotoxin produced by cone snails, conotoxin, has great therapeutic potential in the discovery of new analgesics to treat visceral pain associated with irritable bowel syndrome and inflammatory bowel disease because it is a selective toxin for the N-type voltage-gated calcium channels, which are among the most important molecular modulators of visceral pain [11].

However, most of the attention has been paid to toxins of cyanobacteria because cultivation of cyanobacteria for drug discovery is less expensive compared with other microorganisms. Cyanobacteria are a group of Gram-negative photoautotrophic prokaryotes that can produce diverse secondary metabolites, including lipopeptides, amino acids, macrolides, amides, among others. This variety of compounds leads to a wide range of bioactivities, such as antibacterial, antifungal, antiviral, and anticarcinogenic [12]. For example, microcystin-LR is a cyclopeptide produced by a wide variety of cyanobacteria. It has been shown to target pancreatic cancer cells overexpressing the organic anion transporting polypeptides 1B1 and 1B3, inhibiting cancer proliferation [13].

Dinoflagellates are unicellular planktonic microalgae that have also been the subject of several studies. They produce a wide range of natural biotoxins, some of which are common to cyanobacteria, such as saxitoxin, having unique and valuable potential for the development of new drugs [14]. For example, saxitoxin acts as a sodium channel blocker, preventing the influx flow of sodium ions. It has been shown to have anesthetic properties [15], which could be used for several days to block the sciatic nerve as a pain treatment with low cytotoxicity [16]. Brevetoxin is a biotoxin that has been previously shown to promote neural repair after ischemic stroke in mice by enhancing dendritic arborization, synapse density, and motor recovery [17]. Pectenotoxin-2 is another relevant toxin produced by dinoflagellates that could be valuable as a chemotherapeutic agent, as it leads to depolymerization of actin filaments and activates an intrinsic pathway of apoptosis in p53-deficient tumor cells [18], while it has also been shown to be more effective against cancer cells than against normal cells of the same tissue [19]. Gymnodimines are a type of macrocyclic imine toxins that can act as cholinergic antagonists and have been shown to decrease the accumulation of intracellular amyloid beta-peptide and hyperphosphorylated forms of tau protein in cortical neurons in vitro, suggesting a potential pharmaceutical approach in Alzheimer’s disease [20]. Other reports indicate that gymnodimines have the potential to sensitize a neuroblastoma cell line to the apoptotic effects of okadaic acid, another algal toxin [21].

**Table 1:** Classification of common aquatic biotoxins, their toxicity, and frequently observed adverse symptoms in human poisoning.

Biotoxin	Chemical Structure	Source	Toxic Syndrome	Toxicological Effects	Ref.
Actinoporins	Peptide	Anemones, jellyfish	-	Severe pain, hypotension, and cardiac irregularities	[22,23]
Amphidinolides	Polyether	Dinoflagellates	-	Cytotoxicity	[24,25]
Azaspiracid	Polyether	Dinoflagellates	AZP	Gastrointestinal and neurological symptoms	[26]
Brevetoxin	Polyether	Dinoflagellates	NSP	Gastrointestinal and neurological symptoms, respiratory problems, and muscular pain	[27]
Ciguatoxin	Polyether	Dinoflagellates	CFP	Gastrointestinal, neurological, and cardiovascular symptoms	[27]
Conotoxin	Peptide	Cone snails	-	Muscle paralysis of the diaphragm, and alteration of blood pressure	[7,28]
Dinophysistoxins	Polyether	Dinoflagellates	DSP	Gastrointestinal symptoms	[9]
Domoic acid	Cyclic amino acid	Diatoms	ASP	Gastrointestinal and neurological symptoms	[29]
Gambieric acid	Polyether	Dinoflagellates	-	Cytotoxicity	[30,31]
Gambierol	Polyether	Dinoflagellates	CFP	Gastrointestinal disturbances and neurological alterations	[32]
Goniodomin A	Polyether	Dinoflagellates	-	Hepatotoxicity	[33]
Gymnocin	Polyether	Dinoflagellates	-	Cytotoxicity	[14,34]
Gymnodimine	Cyclic imine	Dinoflagellates	-	Neurological symptoms	[35,36]
Karlotoxin	Polyether	Dinoflagellates	-	Cytotoxicity	[37,38]
Maitotoxin	Polyether	Dinoflagellates	CFP	Neurological symptoms	[27]
Microcystin	Peptide	Cyanobacteria	-	Hepatotoxicity	[39]
Okadaic acid	Polyether	Dinoflagellates	DSP	Gastrointestinal symptoms	[14]
Palytoxin	Polyether	Zoantharians and dinoflagellates	-	Gastrointestinal, cardiac, and respiratory problems	[2,14,40]
Pectenotoxin	Polyether	Dinoflagellates	DSP	Gastrointestinal symptoms and hepatotoxicity	[41]
Saxitoxin	Alkaloid	Dinoflagellates and cyanobacteria	PSP	Gastrointestinal symptoms and respiratory paralysis	[14]
Spirolides	Cyclic imine	Dinoflagellates	-	Neurological symptoms	[35,42]
Tetrodotoxin	Alkaloid	Pufferfish and other species (e.g., starfish, gastropods, newts, and crabs)	-	Neurological symptoms	[8]
Yessotoxin	Polyether	Dinoflagellates	DSP	Gastrointestinal symptoms	[43]

Yessotoxin is considered one of the most polar lipophilic toxins due to the presence of two sulfate groups. This toxin elicits several effects, such as modulation of intracellular calcium and cyclic adenosine monophosphate levels, caspase activation, fragmentation of E-cadherin, and alteration of the cytoskeleton [14]. Considering its pharmaceutical advantages, it has been shown to interfere with the apoptotic pathways of cancer cell lines, inhibiting melanoma tumor growth and causing strong toxicity to a lymphocytic leukemia cell line [44]. Yessotoxin can also cause genotoxicity and induce mitotic catastrophe, followed by cell death, including apoptotic and necrosis-like manner, indicating great potential to control tumor progression [45]. Another toxin isolated from dinoflagellates with interesting properties is amphidinol, which has antifungal and hemolytic activities [46]. A potent neurotoxin found primarily in pufferfish, tetrodotoxin is a selective blocker of voltage-gated sodium channels and has been administered intramuscularly to patients with cancer-related pain, showing successful results with mild secondary effects when present [47,48]. In addition, tetrodotoxin has been shown to alleviate symptoms in patients undergoing heroin withdrawal [49].

Unfortunately, there are still many difficulties in the thorough research of aquatic biotoxins and their use in the development of new drugs with higher efficacy, safety, tolerability, and convenience compared with existing drugs [50]. It is estimated that it takes about 14 years from the identification of the active compound and determination of its functionality to its approval in the market, which becomes very expensive and time-consuming. For this reason, it is necessary to implement new drug discovery alternatives that are more efficient, accurate, less expensive, and faster [51].

Biosensors are characterized by high sensitivity, robustness, speed, selectivity, ease of use, and cost-effectiveness. In particular, optical techniques have experienced significant growth in the research community, mainly because they enable low-cost miniaturized systems with reliable and fast responses. Therefore, optical biosensors have become an excellent method for detecting biological systems and are promoting development in drug discovery, clinical diagnosis, environmental monitoring, and other fields [52].

In this review, after introducing common aquatic-derived biotoxins that have been the subject of research as hazardous compounds but also as promising drugs for medicinal purposes, the focus is to provide a comprehensive article on the progress in optical biosensing for the detection of aquatic biotoxins and the technological advances that will enable the rapid development of a new generation of drugs. First, a brief description of the conventional analytical methods that have been used to date for the detection of aquatic bioactive compounds is given. Then, the advantages and importance of replacing conventional methods with optical biosensors are discussed. Their improved performance, miniaturization, and rapid response improve the detection and monitoring of biotoxins and also contribute to the identification of various biomolecules of clinical interest for new drug development. Interesting examples from the literature of the successful use of optical biosensors for these purposes are presented. Another focus is high-throughput screening (HTS) based on optical biosensors used to screen large compound libraries, especially label-free detection systems. It is expected that these technologies can be used to accelerate biomedical research in the field of bioactive natural products.

## 2. Conventional Methods to Detect Bioactive Natural Compounds

As mentioned above, it is imperative to actively monitor and detect aquatic biotoxins in order to avoid public health risk, contamination of various foods, and potential economic impact, in addition to identifying novel compounds useful for the development of effective drugs. These have the potential to fill the current gaps in pharmacological drugs with anticancer, anti-inflammatory, antifungal, or antibacterial properties without showing toxicity as a side effect. Conventional methods for the detection of biotoxins include animal- and cell-based assays, chemical methods (e.g., liquid chromatography (LC), high-performance liquid chromatography (HPLC), mass spectrometry (MS), and tandem mass spectrometry (MS/MS)), biochemical techniques such as immunoassays, and receptor-based methods [53,54].

### 2.1. Bioassays

The mouse bioassay (MBA) has been the most common bioassay method. In the MBA, mice are injected intraperitoneally with the sample extract to be tested for lethality. Although animal testing is still used worldwide for research purposes, the MBA has been replaced by alternative detection methods for screening lipophilic marine biotoxins (LMBs) and protecting public health [55]. This is due to ethical concerns, as well as high rates of false-positive and false-negative results, low sensitivity, and reduced reproducibility [55,56,57]. Alternative in vitro cell-based techniques, combined with chemical methods, have been investigated for the detection of LMBs [56]. For example, the neuro-2a assay uses murine neuro-2a neuroblastoma cells to determine cell viability upon exposure to LMBs, such as okadaic acid, dinophysistoxins, pectenotoxin-2, azaspiracids, and yessotoxins. Subsequently, this qualitative screening method is complemented by chemical analysis using LC–MS/MS as a reference method [56,58].

### 2.2. Chemical Assays

Separation of samples and their chemical analysis by HPLC is the most widely used method for detecting biotoxins because it is a sensitive, reliable, easy-to-calibrate, and accurate method. It also has the great advantage that it can be coupled with various detection systems, such as fluorescence detection and MS [1]. MS measures the mass-to-charge ratio of molecules present in the sample, allowing rapid analysis with high selectivity, a broad spectrum of samples, and identification of analogues of a particular toxin group. Recently, with the continuous advances in coupling LC and MS techniques, the need for toxin standards and derivatization reagents can be eliminated [1]. This is important because, for example, there are at least 24 saxitoxin analogues and 90 yessotoxin analogues, making it incredibly difficult to have reference materials for all of them [56]. Thus, LC–MS methods have been successfully used to detect various lipophilic toxin classes [57,59,60]. The sample can also be pretreated (e.g., with solid-phase extraction), which allows purification and preconcentration of analytes retained on a sorbent cartridge [1].

### 2.3. Biochemical Assays

An immunoassay is a biochemical assay based on the immunological affinity between an antibody and its antigen. The most commonly used immunoassay for the detection of biotoxins is the enzyme-linked immunosorbent assay (ELISA), in which binding between the biotoxin and its specific antibody is detected based on labeling with an enzyme that converts target recognition into a color reaction upon substrate catalysis. Commonly used labeling enzymes are horseradish peroxidase (HRP) and alkaline phosphatase. The main advantages of ELISA are the simple mechanism, accuracy, and easy equipment operation [1]. Examples of ELISA applications include the detection of yessotoxins in shellfish and algal samples [61], gonyautoxin (a PSP toxin) [62,63], and azaspiracid [64] from shellfish.

In the last decade, new designs of ELISA assays have appeared. For example, Zhang et al. (2012) developed a capillary electrophoresis-based immunoassay with electrochemical detection for saxitoxins in shellfish samples. After a competitive immunoreaction, capillary electrophoresis enabled the separation of the HRP-labeled antibody–antigen complex from the unbound labeled antigen, and then the HRP reaction was followed electrochemically [65]. Kim et al. (2015) prepared a lab-on-a-chip for the detection of saxitoxin using a competitive immunoassay. The biotoxin was captured by functionalized magnetic particles in a sample chamber where the sample and reactants were added. Subsequently, the solid-phase magnetic particles containing the bound toxins were magnetically conducted through the liquid-stationary phase into the detection chamber containing the HRP substrate [66]. Pelin et al. (2018) were able to conjugate cell-based assays with immunoenzymatic detection for the quantification of palytoxin. The biotoxin binds with high affinity to several cell lines and could then be detected by the addition of a specific antibody [67].

Despite advantages in simplicity, sensitivity, and selectivity, immunoassays are still associated with high laboratory and antibody preparation costs, antibody instability, and the possibility of false-positive or false-negative results [68].

## 3. Optical Biosensors for Detecting Aquatic Biotoxins

**Figure 1:**
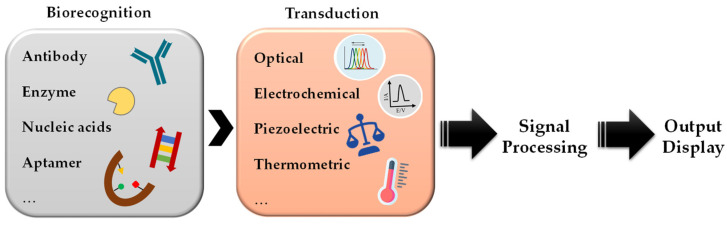
Schematic representation of a biosensor.

Although the traditional analytical methods described so far have a good detection success rate, there is a need for alternative methods that are cheaper, more sensitive, and faster and can be used as a screening tool to evaluate multiple samples in a reasonable amount of time. Biosensors are an alternative to these classical methods as they can detect a wide range of biotoxins in a sensitive and selective manner and can be used as portable and rapid techniques without the need for qualified personnel [69]. A biosensor is an analytical device consisting of a biorecognition element in conjunction with the transducer responsible for converting the recognition reaction into a measurable signal [70] (Figure 1). In this way, biosensors can be classified according to the bioreceptor element and the transducer type. In the first case, they can be classified as immunosensors, aptasensors, enzymatic sensors, nucleic acid sensors, and cell-based sensors [70]. Biosensors can also be categorized by physicochemical signal transduction, with electrochemical, optical, thermal, and piezoelectric sensors being the most common [71].

Although optical and electrochemical biosensors have the highest sensitivity and selectivity compared with the other methods mentioned, only the optical methods are considered in this work. Optical transducers involve a change in absorption, emission, transmission, scattering, reflection, or refraction of light that is proportional to the concentration of the target analyte. In addition, the optical change can be monitored with a label (e.g., a chromophore or fluorophore) or without a label, in which case they are referred to as label-free biosensors [72]. Optical biosensors have experienced a considerable growth for medical diagnosis, food quality control, and environmental monitoring, primarily because they can be designed as low-cost miniaturized systems with reliable and rapid responses [73]. There are many optical approaches, including colorimetric, photonic, fluorescent, surface-enhanced Raman spectroscopy (SERS), surface plasmon resonance (SPR), interferometers, and microresonators. The most appealing examples of the application of these optical methods for the detection of biotoxins are presented below and summarized in Table 2.

### 3.1. Colorimetric Biosensors

Colorimetric strategies for the detection of aquatic biotoxins mainly resort to metal nanoparticles and the change in their aggregation state in the presence of the bioanalyte, as well as the inhibition or activation of an enzyme in the presence of the target, both of which result in a color change. For example, microcystin-LR was successfully detected using specific aptamers as linkers for gold nanoparticle (AuNP) dimers. When the biotoxin was present, the aptamer changed its structure to bind the target, disassembling the dimer. As a result, there was a color shift from blue to red [76]. Li et al. (2016) also detected microcystin-LR, reaching a limit of detection (LOD) of 0.37 nmol L^−1^. In this work, the aptamer binds the AuNPs and protects them from aggregation. Since the aptamer binds the target microcystin-LR with high affinity upon sample loading, there is a displacement of the aptamer, causing the AuNPs to aggregate, resulting in a color change from red to blue [77]. A similar principle using AuNPs and a specific aptamer that reacts with saxitoxin allowed its detection, with a LOD of 10 fmol L^−1^, through aggregation of AuNPs and shift in color to blue [82].

**Figure 2:**
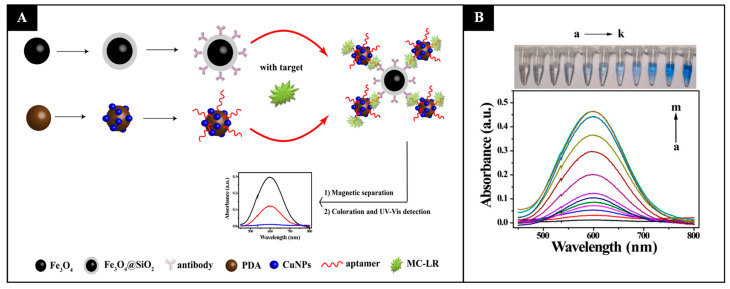
(**A**) Schematic representation of microcystin-LR detection using a colorimetric method based on magnetic separation and copper nanoparticle conversion to Cu^2+^ ions, which then react with bis(cyclohexanone)oxaldihydrazone. (**B**) This reaction gives rise to a blue solution, whose color intensity increases with the concentration of microcystin-LR (nmol L^−1^): (**a**) 0, (**b**) 0.05, (**c**) 1.0, (**d**) 2.0, (**e**) 3.0, (**f**) 4.0, (**g**) 5.0, (**h**) 10.0, (**i**) 15.0, (**j**) 20.0, (**k**) 25.0, (**l**) 30.0, and (**m**) 35.0. (Reproduced under the terms and conditions of Creative Commons Attribution (CC BY) license [78]).

Recently, Tang et al. (2019) achieved a lower LOD of 0.05 nmol L^−1^ for microcystin-LR using a different strategy: antibody-functionalized silica-coated magnetic nanoparticles (Fe_3_O_4_@SiO_2_) and aptamer-functionalized polydopamine nanospheres decorated with copper nanoparticles (PDA/CuNPs) were developed. Here, in the presence of microcystin-LR in the samples, both nanoparticles were bound in sandwich-like composites and could be magnetically separated. Subsequently, the copper was converted to Cu^2+^, which reacted with bis(cyclohexanone)oxaldihydrazone, producing color and allowing a quantitative detection of the toxin [78] (Figure 2).

When colorimetric detection is triggered by an enzymatic reaction, the tests may rely on the inhibition of protein phosphatases or phosphodiesterases [79,80,83]. For example, Hayat et al. (2012) used the inhibition of phosphatase 2A in the presence of okadaic acid, which hindered the hydrolysis of p-nitrophenyl phosphate and prevented the formation of the yellow p-nitrophenol [80]. Other colorimetric immunoassays used glucose oxidase (GOx), AuNPs, and blue staining of oxidized 3,3′,5,5′-tetramethylbenzidine (TMB) [74,75]. Lai et al. (2016) presented a technique based on an enzyme-triggered Fenton reaction. In this work, a competitive immunoassay was performed using nanogold labeled with GOx and an antibody for brevetoxin B. When the target brevetoxin B was present, it competed with the immobilized brevetoxin B on magnetic beads for the labeled antibody. After magnetic separation, the carried enzyme oxidized the glucose, forming hydrogen peroxide, which later oxidized iron (II) to iron (III), also forming the radical hydroxyl; and finally, the resulting iron (III) and the radical oxidized TMB, forming a blue product. The absorbance decreased with an increasing concentration of sampled brevetoxin B [75]. In another study, okadaic acid was detected based on a direct competitive enzyme-linked aptamer assay (ELAA). The aptamer was first immobilized on a microplate and hybridized with the complementary sequence labelled with catalase. In the absence of okadaic acid, catalase consumes hydrogen peroxide. As the concentration of hydrogen peroxide decreases, the red solution of gold trichloric acid turns blue due to aggregated nanoparticles. However, in the presence of okadaic acid, the complementary sequence is replaced, resulting in a high concentration of hydrogen peroxide and a nonaggregated red solution of AuNPs [81].

### 3.2. Fluorescent Biosensors

Fluorescence strategies have been highly studied in the field of biotoxin monitoring. Many examples include the use of indirect detection by competitive assays [85,86,88,89,93], quenching of the fluorescent signal [87,90,127], sandwich assays [84], and aptamer binding [92]. Fluorescent biosensors have been developed not only for single detection but also for multiplex detection. For example, Bickman et al. (2018) designed a multiplex sensor specific for microcystin and cylindrospermopsin cyanotoxins [89]. Liu et al. (2017) were able to detect up to 32 contaminants in lake waters simultaneously, including microcystin-LR. The sensor was based on an integrated multichannel waveguide-based fluorescent sensor functionalized for different contaminants using an indirect competitive immunoassay [91].

The competitive assays were combined with fluorescence quenching/turn-on signal in a study to selectively and sensitively detect tetrodotoxin. For this purpose, the research team used competitive lateral flow immunochromatographic strips (C-LFICSs). The test line contained quantum dot nanobeads (QDNBs) conjugated to the protein BSA, as well as tetrodotoxin–BSA. The QDs were chosen because they have the advantage of higher signal brightness and stability compared with commercial organic fluorophores. Then, gold nanoflowers labeled with an antibody against tetrodotoxin and the sample were added to the C-LFICS so that they could move through the strip by capillarity. If the sample under analysis did not contain tetrodotoxin, the nanoflowers would bind to the tetrodotoxin on the test line, resulting in fluorescence quenching. In the positive case, the nanoflowers recognized the tetrodotoxin on the sample and did not bind to the tetrodotoxin on the test line, causing the fluorescence to remain on [94]. Gholami et al. (2020) based their sensor on fluorescence resonance energy transfer (FRET) between energy donors, carbon QDs (CQDs), and acceptors (AuNPs) for the detection of maitotoxin with low detection limit [87].

**Figure 3:**
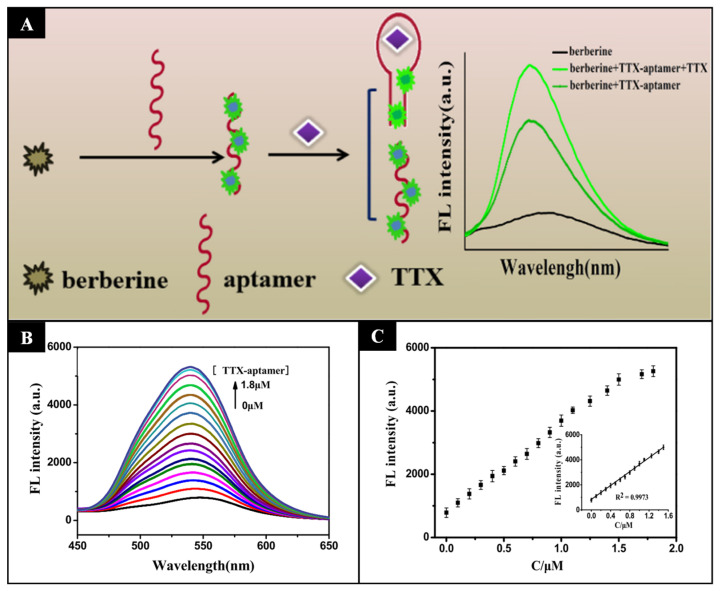
(**A**) Schematic representation of tetrodotoxin detection by a fluorescent method dependent of the aptamer conformational change. (**B**) Fluorescent spectra obtained with different concentrations of tetrodotoxin and (**C**) calibration curve. (Reproduced with permission [95], Copyright 2019, Elsevier).

Lan et al. (2019) chose a different strategy and used a specific nucleic acid aptamer for tetrodotoxin that switches its conformational structure from a single-strand random coil to a compact neck ring structure in the presence of the biotoxin. Therefore, insertion of the fluorophore between the random coil leads to changes in the fluorescence signal due to conformational changes of the aptamer that depend on the concentration of tetrodotoxin. Using this method, a LOD of 0.074 nmol L^−1^ was determined [95] (Figure 3).

### 3.3. Surface-Enhanced Raman Scattering (SERS) Biosensors

SERS is a technique for enhancing Raman scattering of analyte molecules adsorbed on or in close proximity to SERS-active surfaces (i.e., rough or nanostructured metal surfaces, often classically gold, silver, or copper). This method offers high specificity and sensitivity and has been used to detect various toxins, such as saxitoxin [97,99,100], tetrodotoxin [102,103], domoic acid [97], okadaic acid, dinophysistoxins, yessotoxin [96], and microcystin-LR [98].

Saxitoxin shows a weak affinity for AuNPs or silver nanoparticles (AgNPs). Therefore, a surface modification on the SERS substrate was used, as is the case of cysteine-modified AuNPs (Cys-AuNPs). This modification, in coordination with a new method called dynamic SERS, in which self-assembly of nanoparticles is induced by solvent evaporation, increased the available 3D trapping wells for the biotoxin, resulting in a LOD of 0.1 µmol L^−1^ [101]. However, it showed lower sensitivity compared with the first reported SERS method for saxitoxin detection, developed by Pearman et al. (2008), who used a colloidal hydrosol of AgNPs as SERS substrate [99]. The sensitivity increased by combining SERS with laser optical tweezers Raman spectroscopy (LTRS) and reached a LOD of 2 nmol L^−1^. These results arise from the fact that laser optical tweezers are more efficient in capturing numerous AgNPs adsorbed on the saxitoxin molecule, thus enriching the Raman signal [100].

**Figure 4:**
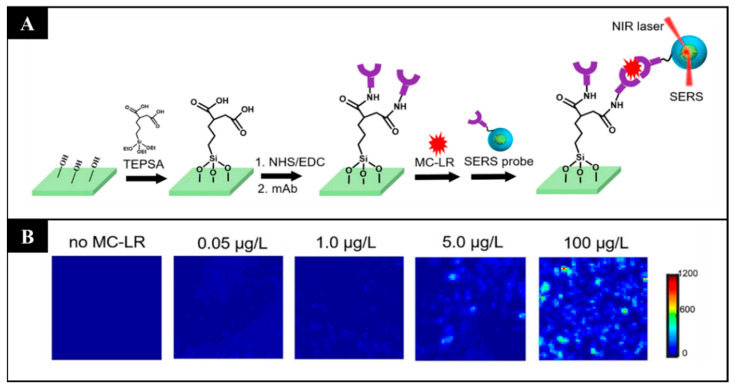
(**A**) Schematic representation of microcystin-LR (MC-LR) detection by an immunosensor and SERS probe. (**B**) Representative SERS images for increasing concentrations of the biotoxin. (Reproduced with permission [98], Copyright 2019, American Society of Chemistry).

Another interesting study is that of Li et al. (2019), who constructed a biosensor that is more sensitive and has a wider detection range than conventional ELISA kits for the detection of microcystin-LR in aquatic environments. In this work, the authors designed particles with a core of plasmonic gold nanostars with Raman reporter molecules (4-nitrothiophenol) embedded between the core and a protective silica shell. The shell improves the stability and reproducibility of the sensor, and the SERS tags have immobilized antibodies against microcystin-LR for specificity [98] (Figure 4).

### 3.4. Surface Plasmon Resonance (SPR) Biosensors

The SPR optical signal results from the oscillation of conduction band electrons at the dielectric–metal interface induced by incident light. Binding of analytes on or near the metal surface leads to differences in the refractive index in the immediate vicinity of the metal surface and enables label-free, real-time detection of intermolecular interactions [107,108]. Gold is the metal that is commonly used in SPR. This optical method has been widely explored for the detection of biotoxins in water and food samples: domoic acid [107,108,109], okadaic acid [111,112], palytoxin [113,114], tetrodotoxin [115,116,117], yessotoxin [118], microcystins [110], and multiple toxins simultaneously [104,105,106,128]. When SPR is used for small molecules of low molecular weight, such as toxins, it is generally associated with amplification steps [129], competition-based assays (where the analyte in solution prevents antibody binding to the analytes immobilized on the surface), or displacement-based assays (where antibodies bound to the immobilized analytes on the SPR surface are displaced by the analyte in solution) [107]. However, Yakes et al. (2014) demonstrated the first direct SPR immunosensor for biotoxin detection, tetrodotoxin in a pufferfish matrix as a proof of concept. The antitetrodotoxin was immobilized on the sensor chip, and the analyte was directly injected into the SPR sensor surface, achieving a LOD of 2 ng mL^−1^ in the pufferfish matrix [117].

There are several approaches to the design of this type of biosensors. For example, Garibo et al. (2014) used magnetic particles functionalized with an antibody against okadaic acid as immobilization supports and carriers of the biotoxin for competitive assay on the SPR immunosensor [112]. Moreover, Stevens et al. (2007) developed a portable six-channel SPR biosensor for the measurement of domoic acid in phosphate-buffered saline and clam extract solutions, reaching a LOD of 10 nmol L^−1^ [107]. Nevertheless, multiplex analysis is of great importance because samples are usually complex and do not contain only one biotoxin. Microfluidic devices allow not only compartmentalization of the sensor to immobilize the target and reversal of the association event to reuse the biosensor, but also multiplex analysis. For example, Campbell et al. (2011) constructed a biosensor with four flow cells, each with four SPR sensing spots, which allowed simultaneous readout of 16 different interactions at the SPR surface. The biosensor also had a liquid handling system for sample injection and regeneration of the sensor for further measurements [105].

### 3.5. Interferometric Biosensors

**Figure 5:**
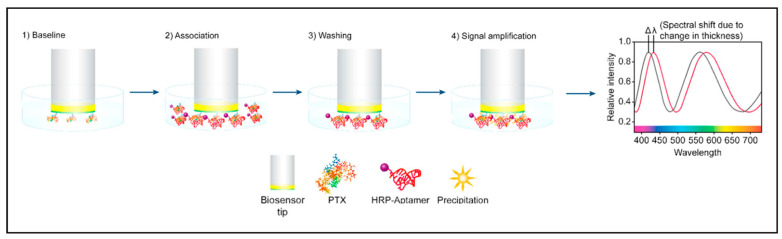
Schematic representation of a competitive biolayer interferometric biosensor for palytoxin (PTX) detection using HRP-labeled aptamers as biorecognition receptors. (Reproduced with permission [123] Copyright 2017, Elsevier).

Interferometry is an optical technique that measures the interference pattern of light produced by two light beams, the sensor where the bioconjugation event occurs and the reference beams. Many interferometric configurations have been used for highly sensitive real-time detection of small molecules [130]. Chocarro-Ruiz et al. (2017) created an immunosensor chip based on bimodal waveguide interferometry. The sensor was functionalized with an antibody against okadaic acid and showed a LOD of 0.2 µg L^−1^ [122]. An interesting approach based on a Fabry–Pérot interferometer was developed by Queirós et al. (2011), who grew a sol–gel molecularly imprinted polymer (MIP) into the tip of the optical fiber by dip-coating, creating a selective membrane for microcystin-LR. The MIP showed a low thermal effect, good for field applications, and excellent selectivity against other coexisting species in the sample [121]. In biolayer interferometry (BLI), the tip of the fiber is coated with a layer of immobilized biomolecules, and the interference pattern of white light reflected from the biolayer and an internal reference surface is analyzed. Any change in the molecules bound to the tip, resulting from analyte recognition, causes a shift in the interference pattern. The wavelength shift is directly related to the distance between the two reflecting surfaces (i.e., it correlates with changes in the thickness of the biolayer resulting from analyte detection) [120]. This technique has been successfully used for the detection of domoic acid [120], palytoxin [123], saxitoxin [124], and dinophysistoxin-1 [119], with low LODs. In the case of palytoxin detection, the signal was amplified by HRP-labeled aptamers specific for palytoxin immobilized on the biosensor surface. HRP aptamers were used as biorecognition receptors to bind competitively with immobilized palytoxin. When the palytoxin/HRP aptamer complex was introduced into a 3,3′-diaminobenzidine solution, a polymeric product precipitated directly in the tip and caused a strong change in the shift of light [123] (Figure 5).

### 3.6. Resonant Mirror (RM) Biosensors

An RM biosensor is a waveguide-based sensor that uses the evanescent field produced at the sensing surface to follow specific interactions between molecules through changes in the refractive index at the sensing surface. These biosensors enable real-time quantitative measurements [131,132]. To our knowledge, there are only two studies using MR to detect aquatic biotoxins. In summary, the authors investigated the affinity between yessotoxin and various phosphodiesterases, a known target of this toxin, which ensures the specificity of the biosensor. The RM biosensor had aminosilane surfaces to immobilize phosphodiesterases, and increasing concentrations of the biotoxin were added, resulting in a proportional increase in sensor response [125]. This sensor approach also allowed the investigation of the specificity of different phosphodiesterase families for yessotoxin [126].

## 4. Optical High-Throughput Screening (HTS) Assays for Drug Discovery

Interest in naturally occurring compounds from aquatic organisms with potent pharmacological activity has long led to research efforts. Indeed, isolated compounds from extracts of marine organisms have shown interesting biological activities beyond their known toxicological effects [133,134]. There are many relevant examples of such bioactive natural compounds with antibacterial, anti-inflammatory, antimalarial, and anticancer activities [135,136]. HTS assays are key processes in drug discovery and consist of automated screening of large compound libraries and identification of biologically relevant compounds. Testing a large number of natural or synthetic chemical compounds for a specific biological target is the starting point of a drug design and development pipeline. The advantages of HTS technology are mainly in reducing the cost of drug development and increasing the speed, simplicity, and process efficiency [137]. HTS assays are divided into biochemical assays or cell-based assays. The first group usually relies on enzyme activity or receptor–ligand binding tests, which allow for obtaining highly reproducible miniaturized assays. However, tissue-specific responses may differ from those in biochemical assays because the activity of a small molecule may be different in a cellular context [138]. Therefore, drug screening is evolving into in vitro cell-based assays that are more suitable in the process of validation of new drugs in the preclinical phase. It offers the possibility to study the toxicity of a given drug for both targeted cell populations and nontargeted cells, allowing the selection of potential drugs without harmful side effects [139].

In the techniques used to study biomolecular interactions and the binding of a ligand to its receptor, the labeling steps required in most methods (e.g., fluorescence labeling and radiolabeling) led to some drawbacks involving additional time and cost. In addition, the labels may interfere with the site of molecular interaction, leading to false-negative results, or bind to the background, leading to false-positive results [140]. In this context, there is a growing awareness of novel label-free optical techniques that provide improved data on interaction specificity, kinetics, and affinity in real time. Moreover, their value already extends beyond low-throughput analysis of binding affinity and kinetics, as newly developed optical biosensor arrays for multiplexed detection offer a greater degree of flexibility in experimental design [140]. Optical biosensors have accompanied the shift in drug discovery from a target-directed approach to a systems biology-centered approach by potentiating the development of cell-based biosensors [141]. Among the label-free screening systems, SPR, RM, interferometry, Raman spectroscopy, and photonic crystal (PC)-based biosensors are some of the best-developed methods for HTS applications with the goal of accelerating the drug discovery process [140,141,142,143,144].

**Figure 6:**
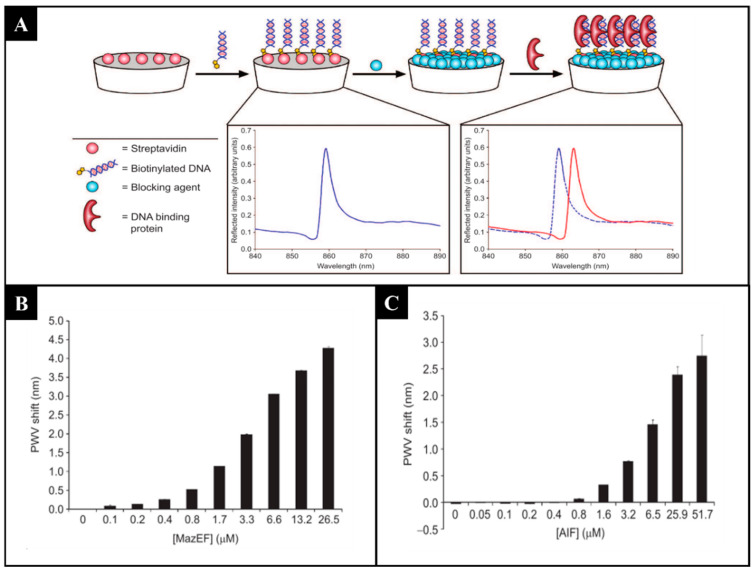
(**A**) Example of an HTS based on photonic crystals for the study of protein–DNA interactions, where the peak wavelength value (PWV) is measured, in particular for different concentrations of (**B**) toxin–antitoxin chromosomal module of *Escherichia coli* (MazEF) and of (**C**) apoptosis-inducing factor (AIF). (Reproduced with permission [152], Copyright 2008, American Society of Chemistry).

Among the various mentioned label-free methods, PCs show a great potential for incorporation into HTS [145]. Biosensor platforms using PCs present many advantages, such as high sensitivity, flexibility in structural design, cost-effective fabrication with a variety of materials, short testing time, and ability to detect a wide range of analytes [146,147]. PCs are one-, two-, or three-dimensional periodic arrays composed of materials with different refractive indexes. The structural color observed in PCs is explained by the photonic band gap (PBG), where certain wavelengths of light do not propagate through the PC and are reflected. When the PBG is in the visible light range, the PC exhibits unique, vibrant colors. These nanostructures occur in nature (e.g., the bright gold and silver colors of jewel scarabs [148] and the blue color of the wings of *Morpho* butterflies) [149]. Bioinspired nanostructured materials exhibiting photonic properties and structural colors have been fabricated for various purposes by controlling composition, additives, and arrangement, among others [150]. Proving its usefulness in HTS, the ability of PCs to identify modulators of protein–protein interactions [151], to discover inhibitors of protein–DNA interactions [152] (Figure 6), and to measure antibody–antibody binding [153] has been demonstrated. Another interesting example of the use of PCs for HTS screening is in cell-based assays. A label-free detection system based on PCs incorporated into a 96-well microplate enabled the quantification of the proliferation of cancer cells and cell apoptosis induced by exposure to a cytotoxic compound [154]. The applications are vast because a variety of cell-based assays can be performed in response to chemical and biological stimuli, while cells are quantitatively monitored in their culture environment over time without the use of dyes or stains [145].

## 5. Conclusions and Future Perspectives

The increasing demand for new detection methods for bioactive natural products, including biotoxins to protect human health, makes optical biosensors an excellent alternative for the detection of these biomolecules compared with conventional methods, such as ELISA, MBA, LC, and MS. Optical biochemical or cell-based assays overcome ethical concerns and irreproducibility associated with animal testing. In addition, optical biosensors exhibit high sensitivity and rapid responses, have lower cost, and are easy to use, often by untrained personnel. The focus of this review was mainly on biosensors for the detection of aquatic biotoxins, as there are great expectations for new, simpler, miniaturized, and improved methods for the real-time detection of various chemicals in water and aquatic organisms. As for other applications, optical biosensors have been successfully used to detect biotoxins even in complex matrices and have shown high sensitivity and selectivity. In addition, new optical detection designs are very promising for HTS of bioactive compounds, as they offer the distinct advantage of being less time-consuming and are cost-effective methods. Label-free optical biosensor arrays will certainly accelerate new areas of drug discovery, as expected, especially regarding the use of HTS for the discovery of aquatic bioactive compounds, and help decipher their pharmaceutical potential.

## Figures and Tables

**Table 2:** Summary of common optical biosensors applied to detect biotoxins. In cases where more than one biotoxin was studied in the cited article, LOD is linked by symbols to the respective biotoxin.

Optical Technique	Biotoxin	Biosensor’s Properties	LOD	Ref.
Colorimetric	Brevetoxin	GOx/AuNPs/TMB oxidation	0.1 ng kg^−1^	[74]
		GOx/AuNPs/TMB oxidation	0.076 ng kg^−1^	[75]
	Microcystins	Disassembly of AuNPs aggregation	0.05 nmol L^−1^	[76]
		AuNPs aggregation	0.37 nmol L^−1^	[77]
		Reaction of Cu^2+^ and bis(cyclohexanone)oxaldihydrazone	0.05 nmol L^−1^	[78]
		Protein phosphatase 1 inhibition	0.01 ng mL^−1^	[79]
	Okadaic acid	Protein phosphatase 2A inhibition	0.29 ng mL^−1^	[80]
		Direct competitive ELAA	0.01 ng mL^−1^	[81]
	Saxitoxin	AuNP aggregation	10 fmol L^−1^	[82]
	Yessotoxin	Phosphodiesterase inhibition	0.8 µmol L^−1^	[83]
Fluorescent	Ciguatoxins	Sandwich ELISA	1 pg mL^−1^	[84]
	Cyclic imines	Competitive assay	10.2 nmol L^−1^	[85]
	Domoic acid	Competitive immunoassay	-	[86]
	Maitotoxin	Fluorescence quenching of CQDs through FRET between CQDs and AuNPs	0.3 pmol L^−1^	[87]
	Microcystins	Competitive assay	10 pmol L^−1^	[88]
		Competitive immunoassay	0.4 ng mL^−1^	[89]
		Fluorescence quenching of graphene oxide (GO) by FRET between GO and AuNPs to detect microcystin-LR * and microcystin-RR ^§^	* 0.5 ng mL^−1^ ^§^ 0.3 ng mL^−1^	[90]
		Integrated optical waveguide-based fluorescent immunosensor	0.21 ng mL^−1^	[91]
	Saxitoxin	Conformational change of aptamer structure	7.5 ng mL^−1^	[92]
	Tetrodotoxin	Competitive immunoassay	2.5 ng mL^−1^	[93]
		Lateral flow device, based on fluorescence quenching/turn-on signal	0.2 ng mL^−1^	[94]
		Conformational change of aptamer structure	0.074 nmol L^−1^	[95]
SERS	Dinophysistoxins	In situ SERS analysis using AgNPs	-	[96]
	Domoic acid	AgNPs as SERS substrate	0.025 mmol L^−1^	[97]
	Microcystin-LR	SERS immunosensor	0.014 ng mL^−1^	[98]
	Okadaic acid	In situ SERS analysis using AgNPs	-	[96]
	Saxitoxin	AgNPs as SERS substrate	3 nmol L^−1^	[99]
		AgNPs as SERS substrate	170 nmol L^−1^	[97]
		SERS combined with LTRS	2 nmol L^−1^	[100]
		Dynamic SERS with Cys-AuNPs as substrate	0.1 µmol L^−1^	[101]
	Tetrodotoxin	AgNP arrays as SERS substrate	0.9 ng mL^−1^	[102]
		SERS with immunomagnetic concentration	10 ng mL^−1^	[103]
	Yessotoxin	In situ SERS analysis using AgNPs	-	[96]
SPR	Analysis multiplex	SPR inhibition assay for saxitoxins and gonyautoxins in shellfish	-	[104]
		SPR immunosensor for saxitoxin *, neosaxitoxin ^§^, domoic acid ^ǂ^, and okadaic acid ^†^	* 1.0 ng mL^−1^ ^§^ 1.1 ng mL^−1^ ^ǂ^ 1.0 ng mL^−1^ ^†^ 1.7 ng mL^−1^	[105]
		SPR immunosensor for saxitoxin *, okadaic acid ^§^, and domoic acid ^ǂ^	* 0.82 ng mL^−1^ ^§^ 0.36 ng mL^−1^ ^ǂ^ 1.66 ng mL^−1^	[106]
	Domoic acid	Competition-based SPR assay	10 nmol L^−1^	[107]
		Inhibition-based SPR assay	0.1 ng mL^−1^	[108]
		In situ underwater SPR system	0.1 ng mL^−1^	[109]
	Microcystin-LR	SPR competitive inhibition assay	73 pg mL^−1^	[110]
	Okadaic acid	SPR immunosensor for shellfish extracts	126 ng g^−1^	[111]
		SPR immunosensor combined with magnetic particles	2.6 ng mL^−1^	[112]
	Palytoxin	SPR method based on Na^+^, K^+^-ATPase affinity	3.73 pg	[113]
		SPR immunosensor for seafood matrices, 10% grouper * and 10% clam ^§^	* 2.8 ng mL^−1^ ^§^ 1.4 ng mL^−1^	[114]
	Tetrodotoxin	SPR inhibition immunoassay for complex matrices, 10% pufferfish liver extract *, 10% pufferfish muscle extract ^§^, and 10% human urine ^ǂ^	* 1 ng mL^−1^ ^§^ 6 ng mL^−1^ ^ǂ^ 17 ng mL^−1^	[115]
		SPR inhibition immunoassay for complex matrices, 10% pufferfish extract * and 10% skim milk ^§^	* 6.13 ng mL^−1^ ^§^ 5.02 ng mL^−1^	[116]
		SPR immunosensor for pufferfish extract	2 ng mL^−1^	[117]
	Yessotoxin	SPR method based on phosphodiesterase enzymes	^-^	[118]
Interferometric	Dinophysistoxin	Aptamer-based BLI	614 pmol L^−1^	[119]
	Domoic acid	BLI with immobilized domoic acid and competitive immunoassay	-	[120]
	Microcystin-LR	MIP-based interferometer	^-^	[121]
	Okadaic acid	Bimodal waveguide immunosensor	0.2 ng mL^−1^	[122]
	Palytoxin	BLI with immobilized palytoxin and HRP-labeled aptamer competitive assay	0.04 pg mL^−1^	[123]
	Saxitoxin	BLI with immobilized saxitoxin and aptamer-based competitive assay	0.5 ng mL^−1^	[124]
RM	Yessotoxin	RM based on phosphodiesterases	^-^	[125,126]

## Data Availability

Not applicable.
